# Learning your way in a city: experience and gender differences in configurational knowledge of one’s environment

**DOI:** 10.3389/fpsyg.2015.00402

**Published:** 2015-04-10

**Authors:** Maartje De Goede, Albert Postma

**Affiliations:** ^1^Experimental Psychology, Helmholtz Research Institute, Utrecht UniversityNetherlands; ^2^TNO Earth, Life, and Social SciencesSoesterberg, Netherlands; ^3^Department of Neurology, Rudolf Magnus Institute of Neuroscience, University Medical Center UtrechtUtrecht, Netherlands

**Keywords:** configurational knowledge, experience, gender differences, navigation, symbolic distance effect, spatial priming

## Abstract

Males tend to outperform females in their knowledge of relative and absolute distances in spatial layouts and environments. It is unclear yet in how far these differences are innate or develop through life. The aim of the present study was to investigate whether gender differences in configurational knowledge for a natural environment might be modulated by experience. In order to examine this possibility, distance as well as directional knowledge of the city of Utrecht in the Netherlands was assessed in male and female inhabitants who had different levels of familiarity with this city. Experience affected the ability to solve difficult distance knowledge problems, but only for females. While the quality of the spatial representation of metric distances improved with more experience, this effect was not different for males and females. In contrast directional configurational measures did show a main gender effect but no experience modulation. In general, it seems that we obtain different configurational aspects according to different experiential time schemes. Moreover, the results suggest that experience may be a modulating factor in the occurrence of gender differences in configurational knowledge, though this seems dependent on the type of measurement. It is discussed in how far proficiency in mental rotation ability and spatial working memory accounts for these differences.

## Introduction

In order to interact efficiently with the outside world, humans need to acquire a rich variety of spatial knowledge about their daily environment. This accumulation of knowledge starts already early in life, such as when a 3 years-old child is taken for the first time by his mother to kindergarten. The extent to which young children can learn to find their way in the world depends on a process of biological brain maturation, on experience with certain information sources (such as novel environments), and/or on their (possibly bi-directional) interaction (cf. Newcombe et al., 1998; Newcombe and Huttenlocher, 2000, 2006; Bullens, 2009). Neuroimaging studies with normally developing children or with children from clinical populations may provide further insights in the respective roles of brain maturation and experience when growing up (Overman et al., 1996a,b). In the current paper we focused particularly upon the role of experience when learning a new spatial environment.

Abundant evidence suggests that from repeated exposures to new surroundings we manage to construct quite elaborate mental representations (cognitive maps) of spatial relations between places (see for example: McNamara, 1986). The quality of these representations may depend on individual differences. Numerous studies have shown that males tend to outperform females on their configurational knowledge of previously learned environments or spatial layouts (see for example: Silverman et al., 2000; Coluccia and Louse, 2004; Fields and Shelton, 2006; Castelli et al., 2008). An explanation for these findings has been sought in the fact that males have a preference for processing geometrical knowledge, comprehending distances and orientations, as opposed to a more route-oriented and landmark-focused approach in females (Montello et al., 1999; Coluccia and Louse, 2004). Studies in different settings have supported this notion. When knowledge retained from map- or route learning is assessed, females retrieve more landmarks whereas males retrieve more distance and directional aspects (Galea and Kimura, 1993). The same difference in cue-preference is reflected in route descriptions of men and women (Dabbs et al., 1998; Lawton, 2001) and findings also extend to actual navigation (Sandstrom et al., 1998).

In contrast with the majority of evidence on configurational knowledge demonstrating a male advantage, some studies have failed to show any superior performance in males (see: Coluccia and Louse, 2004). A remarkable aspect is that often an everyday, to some extent familiar, environment has been used in these studies. for example, Montello and Pick (1993) did not show any male advantage on a pointing task in a real, at least to some extent familiar, environment. Also Montello et al. (1999) did not find a male advantage on a straight line distance comparison task between landmarks, placed on well-learned routes on a campus site. Based on these observations, several authors have argued (see: Montello et al., 1999; Coluccia and Louse, 2004; Fields and Shelton, 2006) that *experience* (familiarity) might be a key factor in the occurrence of sex differences in tasks assessing configurational knowledge.

In general, the content of configurational representations is assumed to develop over time. In particular, with time more detailed information on distances and directions between places emerges (Golledge, 1999). Since in many studies that assess spatial abilities new (computerized) environments are used, male advantages in configurational knowledge could derive from their initial preference for processing geometric aspects of an environment. Over time with more experience females might become as proficient as males in developing and retrieving such type of knowledge. This viewpoint is further sustained by the fact that in experimental paradigms with artificially constructed, but overlearned, environments, gender differences on tests of configurational knowledge are typically absent (Coluccia and Louse, 2004). For example, in a study by Shelton and McNamara (2004), no gender differences appeared in judgements of relative directions after extensive overlearning of a virtual environment. Fields and Shelton (2006) on the other hand, found a male advantage in judgements of relative directions when learning of a virtual environment was limited.

Only few previous studies have considered familiarity or experience as an important factor in the assessment of (gender differences in) configurational knowledge. Kirasic et al. (1984) assessed configurational knowledge of a university campus, involving direction and distance measures, in freshmen and upperclassmen. Males outperformed females on a pointing task, but only when specific places were included. This might have to do, as the authors themselves suggested, with the more frequent use by males of these buildings. Firm conclusions on this matter could not be given, however, since familiarity with the different places was not assessed. Evans and Pezdek (1980) investigated directional and distance knowledge in participants who were either familiar or unfamiliar with a campus. Participants who were unfamiliar with the used environment learned it from a map, whereas ‘familiar participants’ experienced the environment by active navigation. With this setup, it was unclear whether differences in performance were due to differences in familiarity or in the way the environment had been learned.

In light of the foregoing, the aim of the present study was to provide further insights into the effects of experience in males and females on the construction of mental representations of their daily environment. The use of the latter is particularly attractive because it sheds light on the natural development of our everyday spatial knowledge. In the present study, configurational knowledge of the city center of Utrecht (The Netherlands) was assessed in two groups (both half male) of participants who each had a different degree of experience with the city (number of months being an inhabitant of Utrecht) at the moment of testing. Moreover, participants’ familiarity with the different places used in the tasks, was taken into account. Two different types of configurational knowledge were measured, namely distance knowledge and knowledge of the relative positions of places in the environment, i.e., directional knowledge.

A task which might be particularly suited to address the quality of distance knowledge is the *distance comparison task* (see: Denis and Zimmer, 1992; Noordzij and Postma, 2005; Péruch et al., 2006). Previous studies using this task have shown that when distances have to be compared mentally, after having learned an environment either visually or verbally, response times tend to correlate negatively with the magnitude of the differences between the distances being compared. This so-called ‘symbolic-distance effect’ (Moyer and Bayer, 1976) reflects the extent to which mental representations preserve the actual metric properties of the learned environment. As such, the distance comparison task in the present study provides important information on how the quality of spatial representations unfolds over time and whether this is different for males and females.

Whereas different patterns of performance between individuals and conditions might relate to differences in the characteristics of spatial representations, they might also arise because of the employment of different retrieval strategies. One way to exclude any influences of strategic processes is by employing a so called spatial priming procedure. In previous studies on mental representations it has been shown that when people learn a spatial configuration, places close in space prime one another (McNamara, 1986; Noordzij and Postma, 2005). In light of this, our study started with a task aimed at assessing such a priming effect. In this task participants were instructed to decide as fast and accurately as possible whether a place is situated in the city of Utrecht or not. The rationale behind this setup is that a place preceded by a place nearby is primed stronger (and therefore faster responded to) compared to a place that is preceded by a place relatively further away. This way the effect of experience on the implicit spatial representation of the city of Utrecht males and females possess, supposedly unaffected by conscious choices and strategies, could be assessed.

Besides distances, another important dimension of configurational knowledge concerns global place orientations, i.e., the relative positions of places in terms of direction. Whereas tasks assessing distances have shown quite mixed findings with respect to gender differences, tasks measuring orientation knowledge have indicated a stronger male advantage (Coluccia and Louse, 2004). Apparently, different tests of configurational knowledge can produce distinct results, stemming from different task demands, which in turn can affect the occurrence of individual differences (Kitchin, 1996; Coluccia and Louse, 2004). Therefore, in the present study, both distance knowledge and knowledge of relative positions were taken into account.

In addition to the measures of configurational knowledge, also correlations with certain general spatial abilities were considered. It has been suggested that males excel in active mental manipulation of spatial information, which might induce an advantage in spatial orientation tasks during navigation (Bosco et al., 2004). By also assessing general spatial abilities, we could obtain further insights in the cognitive factors underlying any differences in building and employing an environmental map. Therefore, performance on our configurational tasks was related to two potentially relevant basic spatial skills: mental rotation (MR) and spatial working memory. Previous work has suggested that navigational skills could depend on cognitive abilities such as MR proficience (e.g., Moffat et al., 1998; Malinowski, 2001) and spatial working memory capacity (e.g., Bosco et al., 2004).

## Material and Methods

### Participants

Forty-one right-handed healthy males and 43 right-handed healthy females, with normal or corrected-to-normal vision participated in this study. All participants gave informed consent and received 12 euros or two course credits for participation. Participants were subdivided according to their experience with the city of Utrecht, the Netherlands. Participants either had lived for a minimum of 6 months and a maximum of 12 months (*short*) in Utrecht, or they had lived in Utrecht for more than 36 months (*long*) at the time of the experiment. Further requirements were that participants had to visit the city center of Utrecht at least several times a month (on average they did weekly to daily), whereas they should not have visited the city center more than once a month before they lived in Utrecht. Data from 39 males (mean age: 21.5, SD = 2.8) and 42 females (mean age: 21.6, SD = 2.4) were eventually analyzed. Three participants (two males, one female) exhibited extremely poor performance on one or more tasks and were therefore excluded from further analyses. Of the remaining participants 38 participants (18 males an 20 females) had lived for a short time in Utrecht and 43 participants (21 males and 22 females) had lived for a long time in Utrecht. Overall, males and females did not differ in age, *t*(79) = 0.1, *p* = 0.9, or number of months they had lived in the city of Utrecht, *t*(79) = 0.12, *p* = 0.9.

The current study was approved by the medical ethics committee of Utrecht University.

### Tasks and Procedure

Twelve places, assumed to be generally well known by inhabitants of the city of Utrecht, were used in the Familiarity Rating task, the Distance Comparison task, and the Relative Position task. In the Priming task, only eight of these 12 places were used, since four of the 12 places (two department stores, the city theater and the library) can be found in other cities as well.

All computer tasks were created with E-prime (E-prime 1.1; Psychology Software Tools, Inc.) and were run on a Pentium PC with a 17*^′′^* monitor. A response box was used to collect participants’ choices in the Priming task, the Distance comparison task, the Relative Positions task and the MR Task. The keyboard was used to collect responses in the Familiarity Rating task. Data on a perspective taking task were also collected but are not reported here because of a potential measurement error.

#### Priming Task

The first task was conducted with the aim to test the spatial representation participants possessed of the city of Utrecht, while the participants were unaware of this task objective. The task was presented to the participants as a recognition task, in which they had to decide as fast and accurately as possible whether serially presented places (written) are located in Utrecht or not. A list of places was constructed, with half of the places situated in Utrecht and the other half of the places situated in other Dutch cities. The Utrecht-places were used to assess two priming relations: close in space and far in space. The rationale behind this task is the assumption that places preceded by places close in space are responded to faster than a place preceded by a place far in space. A Utrecht place could either be preceded by a place outside of Utrecht, a place close by in Utrecht or a place far away. Places between 101 and 312 meters apart were defined as close in space, whereas places between 547 and 850 meters apart were defined as far in space. Both priming relations were repeated seven times. Place names were repeated two or three times.

#### Familiarity Rating Task

Before participants conducted the Distance comparison task, they were asked how well they knew each of the 12 places in Utrecht used (*place identity*) and how well they knew where they were located in Utrecht (*place position*), both to be indicated on a scale from 1 ( = very poor) to 5 ( = very well). On every trial, two pictures (from different perspectives) of one place were shown on a computer screen. Participants could indicate both their answers by pressing the appropriate numbers on the keyboard. If participants answered with a three or lower, the experimenter explained the place and its position until it was clear to the participant. Nevertheless, trials in which places were used on which participants answered lower than a three were removed from further analyses in all tasks.

#### Distance Comparison Task

In the Distance comparison task two pairs of places were serially presented on each trial. The participant had to indicate which of the two pairs were closer together in the real world, judging interplace distances from the closest point-to-point distance. The first place-pair was presented for 4 s. After an interval of 1 s, the second pair followed and remained until the subject responded. Participants had to indicate as fast and accurately as possible on a response box whether the second pair represented the shortest (green button) or the longest (red button) distance. The task consisted of 96 trials, half of them starting with the same place in both pairs (e.g., city theater – Dom tower and city theater – cinema), half of them starting with different places in both pairs (e.g., city theater – Dom tower and village square – cinema). The task consisted of three blocks, separated by a short break. Blocks were presented in three different orders, pseudo-randomized between participants. Within blocks, trials were fully randomized. Each place-pair was used four times, in order to create a continuous range of interpair distances. The interpair distance ratio (calculated by dividing the distance between places closest in space by the distance between the other two places), ranged from 0.14 to 0.92.

The Distance comparison task was preceded by an instruction and eight practice trials, containing capital cities in Europe. During the practice trials participants received feedback.

#### Relative Positions Task

**FIGURE 1:**
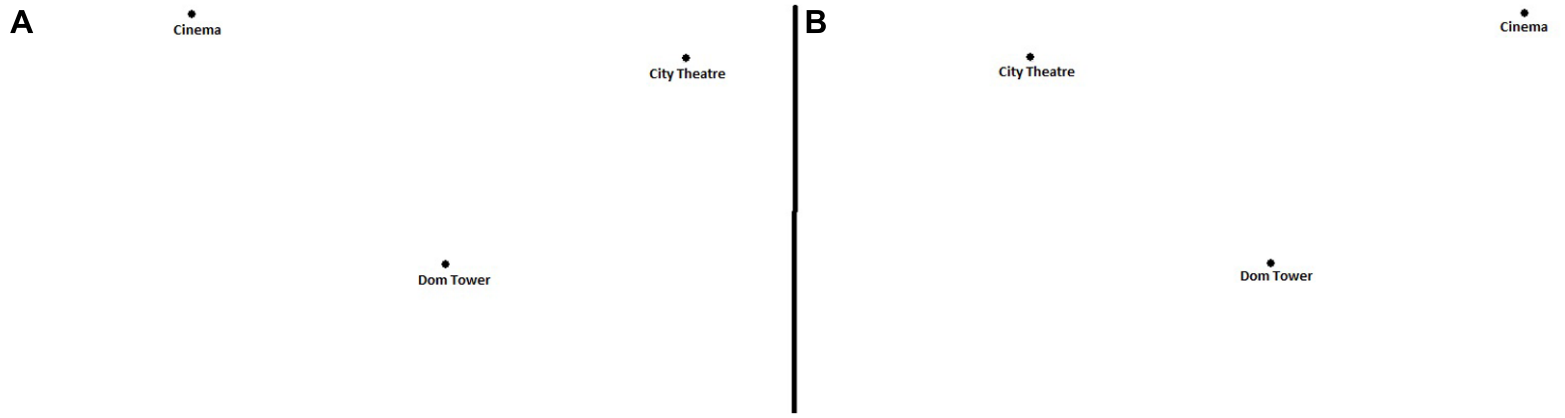
**One correct **(A)** and one mirrored, incorrect **(B)** place triad from the Relative Position judgment task for buildings found in the city of Utrecht**.

In this task participants were shown triads of Utrecht places. They had to judge whether each triad accurately depicted the relative physical position of the places in the real environment (see **Figure 1**). Each place was represented by a black dot with a verbal label below it. Participants were presented 12 different place triads, that were either in the correct relative spatial position or in the incorrect mirror image of the correct spatial position. Each triad was shown at 0, 60, 120, and 180° orientation from the Cartesian coordinates from a standard map. In total, 96 triads were presented to the participants, separated in three blocks with a short break between blocks. Blocks were presented in three different orders, pseudo-randomized between participants. Within blocks, triads were presented in a fixed order, to prevent the occurrence of two or three of the same places following one another. The distance among the three places was always correct to scale.

The Relative Position task was preceded by an instruction and eight practice trials, containing three well known cities in the Netherlands. During the practice trials participants received feedback.

### General Spatial Abilities

Performance on the Distance comparison task and the Position Task was correlated with two general spatial abilities tasks: MR and visuo-spatial working memory.

#### Mental Rotation

Mental rotation was assessed by a computerized version of the MR task designed by Peters et al. (1995), which was based on the original MR task by Vandenberg and Kuse (1978). In this task participants had to decide as accurately and fast as possible whether a rotated blocked figure was the same as a standard figure, depicted besides it, or whether the two figures were different. The task consisted of 48 trials, preceded by an instruction and six practice trials.

#### Visuo-Spatial Working Memory

Visuo-spatial working memory was assessed by the Corsi Block task (Corsi, 1972; Kessels et al., 2000). The test consists of a plastic rectangular plate on which nine cubes are fixed. The experimenter tapped a specific sequence of blocks, which the participant was asked to reproduce. Sequences increased in number of cubes, the longest sequence containing nine blocks. If the participant failed to repeat two sequences in a row, the task was ended.

## Results

### Priming task

**FIGURE 2:**
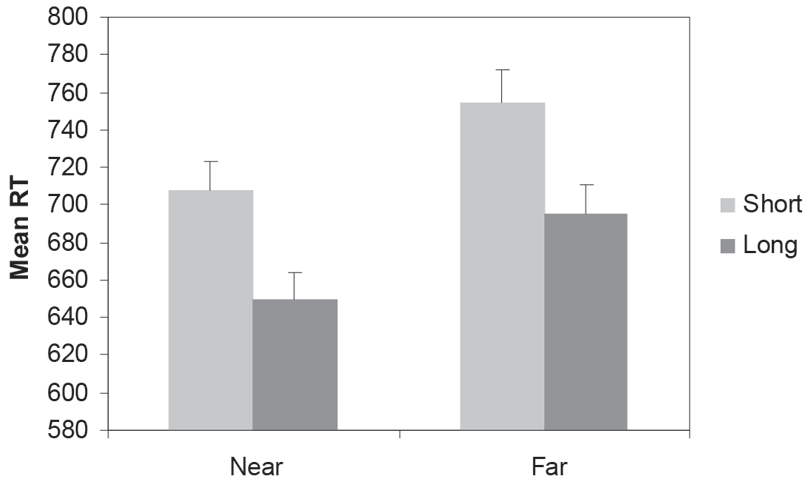
**Mean RTs (+SEM) for the priming/recognition task for near/far priming relations, according to the amount of experience with the city of Utrecht (short/long)**.

For the Recognition/Priming task, mean RT’s for places from the city of Utrecht, computed over correct trials, were analyzed using a 2 × 2 × 2 repeated measures ANOVA with prime-target relation (far/near, separating trials preceded by a far prime from trials preceded by a near prime) as within-subjects variable and gender and experience (having lived for a long/short time in Utrecht) as between-subjects variables. Main effects were found for prime-target relation, *F*(1,77) = 35,04, *p* < 0.01; $\eta_{p}^{2}$ = 0.31, and experience, *F*(1,77) = 7.4, *p* < 0.01; $\eta_{p}^{2}$ = 0.1. RTs for primes and targets near in space were shorter than RTs for prime-targets far in space. Moreover, RTs were shorter for participants who had lived for a long time in Utrecht than for participants who had lived for a short time in Utrecht (see **Figure 2**). No interaction effect between prime-target relation and experience was found, *F*(1,77) = 0.01, *p* = 0.9. Also no main effect of gender, *F*(1,77) = 0.44, *p* = 0.5, nor an inter-action effect prime-target relation × gender, *F*(1,77) = 1.05, *p* = 0.3 or an inter-action between prime-target relation × gender × experience was obtained, *F*(1,77) = 1, *p* = 0.3.

### Familiarity Rating Task

A 2 × 2 ANOVA with gender and experience with between-subjects factors only showed a main effect of experience, *F*(1,77) = 58.2, *p* < 0.01; $\eta_{p}^{2}$ = 0.43. Gender was not significant, *F*(1,77) = 0.35, *p* = 0.56, neither was the interaction gender × experience, *F*(1,77) = 0.79, *p* = 0.38. Participants who had lived for a long time in Utrecht rated the Utrecht place positions as more familiar (mean rating = 4.87, SE = 0.07) than participants who had lived for a short time in Utrecht (mean rating = 4.32, SE = 0.03). Also when place *identity* was added as a covariate, based on the idea that knowledge of a place location is directly related to the knowledge of its identity, this effect remained significant, *F*(1,76) = 5.46, *p* < 0.05; $\eta_{p}^{2}$ = 0.07.

### Distance Comparison Task

Both accuracy (mean proportion correct trials) and RTs were analyzed. RTs were only calculated over correct trials. Trials for which RTs were more than 3 SDs above or below the mean were excluded from analyses. In **Table 1** correlations are shown between overall mean RTs and accuracy scores, and the ratio of each place interpair distance, separated for males and females with short or long experience with the city of Utrecht. Interpair-distance ratios for which mean accuracy was below 0.55, were excluded from analyses. This was the case for interpair-distance ratios of 0.81, 0.88, 0.89, 0.92, and 0.95. All correlations were strongly significant, implying that participants found it harder to compare two distances when the ratio of the interpair distances was larger (i.e., the two pairs were closer in actual distance). This finding clearly demonstrates the presence of the symbolic distance effect.

**Table 1:** Correlation between mean RTs, accuracy and interpair distance ratios.

	Short experience		Long experience	
	Males	Females	Males	Females
RT	0.42^∗∗^	0.43^∗∗^	0.67^∗∗^	0.64^∗∗^
Accuracy	-0.54^∗∗^	-0.47^∗∗^	-0.58^∗∗^	-0.42^∗∗^

^∗∗^is significant with *p* < 0.001.

**FIGURE 3:**
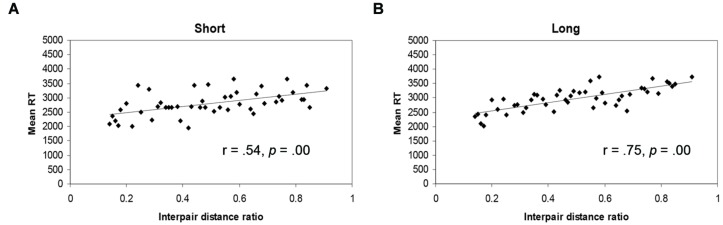
**Mean RTs related to interpair-distance ratios for participants who either had lived for a short **(A)** or a long **(B)** time in the city of Utrecht**.

Subsequently, the distributions of correlations were normalized, using Fisher’s *r*-to-*z* transformations in order to compare their confidence intervals. When short and long experienced groups were compared (one-tailed), independent of gender, a significant difference between correlation strengths for RTs was found [Short: *r*(48) = 0.54; Long: *r*(48) = 0.75; *p* < 0.05; see **Figures 3A,B**]. When considering these correlations within males and females separately, for males a significant increase in correlation strength was demonstrated (*p* < 0.05), whereas for females a nearly significant difference was shown in the same direction (*p* = 0.08; see **Table 1**). Males and females did not differ significantly on the overall symbolic difference effect (*p* = 0.4).

**FIGURE 4:**
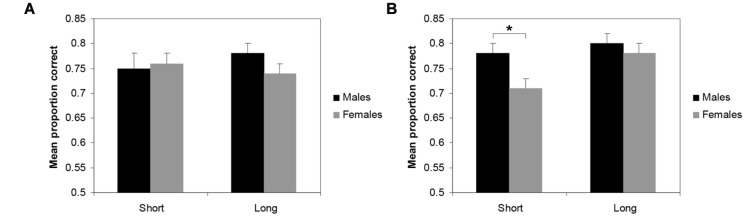
**Mean proportion of correct trials (+SEM) in the distance comparison task for males and females who either had lived for a short or a long time in the city of Utrecht. (A)** performance on distance pairs starting with the same place **(B)** performance on distance pairs starting with a different starting place. *p < 0.05.

For both the RTs and the accuracy scores, A 2 × 2 × 2 repeated measures ANOVA was performed with pair-type (both place pairs starting with the same/different places) as within-subjects variable and gender and experience (short/long) as between-subjects variable. For the RTs, only a main effect of pair-type was found, *F*(1,77) = 16.1, *p* < 0.01; $\eta_{p}^{2}$ = 0.17, indicating that it took participants longer to solve the distance pairs starting with different places than pairs starting with the same place. For the accuracy scores, a three-way interaction was found between pair-type, gender and experience, *F*(1,77) = 4.79, *p* < 0.05; $\eta_{p}^{2}$ = 0.06. *Post hoc* LSD tests, conducted separately for the two sort of trials, showed that only in the trials in which place pairs started with different places, significant group contrasts were observed. In the participants who had lived for a short time in Utrecht, males outperform females (*p* < 0.05). Moreover, females who had lived for a long time in Utrecht outperformed females who had lived for a short time in Utrecht (*p* < 0.05). No main effects or two-way interactions were found. (see **Figure 4**).

### Relative Positions Task

**FIGURE 5:**
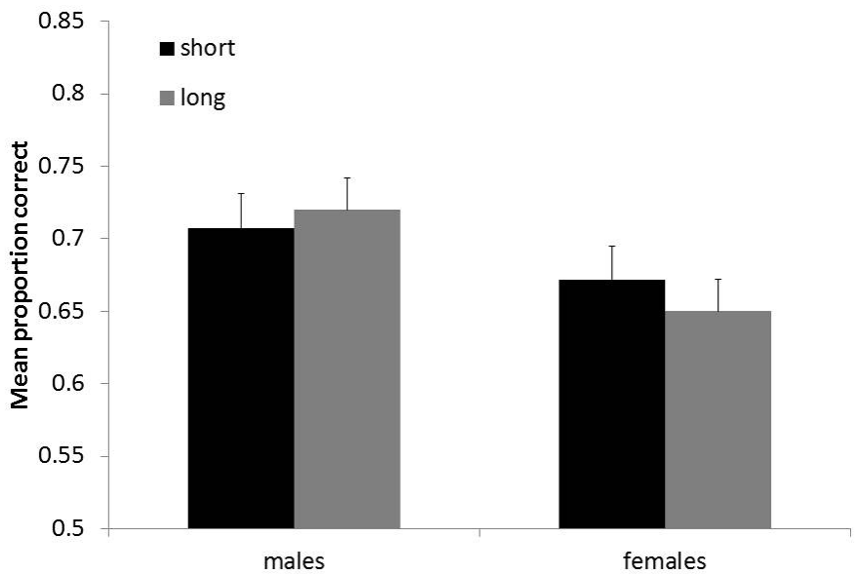
**Mean proportion correct trials (+SEM) in the Position task for males and females**.

Both accuracy (mean proportion correct trials) and RTs were analyzed. RTs were only calculated over correct trials. Trials with RTs more than 3 SDs above or below the mean were excluded from analyses. RTs did not show any effects. An ANOVA carried out with mean proportion correct trials as dependent variable and gender and experience (short/long) as between-subjects variables revealed a significant main effect of gender, *F*(1,77) = 5.4, *p* < 0.05; $\eta_{p}^{2}$ = 0.07, showing that mean performance was higher in males than in females (see **Figure 5**). Experience was not significant, *F*(1,77) = 0.05, *p* = 0.83, nor was the interaction gender by experience, *F*(1,77) = 0.63, *p* = 0.43.

### General Spatial Abilities

In **Table 2** performance on the psychometric tasks is shown for males and females. Scores on the Corsi Block task were computed by the forward block span (CB), equalling the length of the last correctly repeated, forward sequence participants managed to reproduce (maximum = 9).

**Table 2:** Mean performance (+/- SEM) on tasks of general spatial abilities in males and females.

		Males	Females
Mental Rotation	RT (sec)	5.53 (0.26)	6.22 (0.30)
	Accuracy	0.86 (0.01)^∗^	0.81 (0.02)
Corsi Block		6.3 (0.15)	6.3 (0.15)

^∗^is significant with *p* < 0.05.

Only for the MR task a gender difference was observed. Males were significantly faster than females, *t*(79) = 2.57, *p* < 0.05. Accuracy scores showed a non-significant trend. Males had slightly higher accuracy scores, *t*(79) = -1.73, *p* = 0.087.

Correlation analyses were carried out between the two psychometric tasks and the tasks assessing configurational knowledge for males and females, living for a short- or long time in Utrecht (see **Table 3**). All significant correlations signify an increase of performance on the configurational tasks with better performance on the psychometric tasks. For males who had lived for a long time in Utrecht, a positive correlation was found between performance on the more difficult part of Distance comparison task and CB, *r*(21) = 0.55, *p* < 0.05. In the same group performance on the relative positions task showed a significant correlation with CB performance, *r*(21) = 0.46, *p* < 0.05, and MR performance (accuracy), *r*(21) = 0.49, *p* < 0.05. For females who had lived for a short time in Utrecht a significant relation was found between MR performance (RTs) and the easy distance trials, *r*(20) = -0.46, *p* < 0.05.

**Table 3:** Correlation between configurational tasks and psychometric tasks.

	CB		MR			
	Short	Long	Short		Long
			Acc	RT	Acc	RT
**Males**
Distance sa	0.04	0.25	0.19	-0.04	0.28	0.18
Distance dif	-0.17	**0.55^∗^**	-0.23	0.31	0.16	0.26
Relative Position	0.21	**0.46^∗^**	-0.30	-0.28	**0.49^∗^**	-0.02
**Females**
Distance sa	0.07	-0.32	0.24	-**0.46^∗^**	-0.21	0.33
Distance dif	-10	-0.10	0.06	-0.12	-0.1	0.11
Relative Position	-0.03	0.34	0.15	-0.18	0.13	-0.21

Numbers in bold are significant correlations. ^∗^ is significant with p < 0.05. Distance sa, accuracy in comparing distances with same starting place; Distance dif, accuracy in comparing distances with different starting place; CB, Corsi block tapping forward span; MR, mental rotation (both accuracy and reaction times).

## Discussion

In this study we investigated whether and how differences between men and women in configurational knowledge of their daily environment were modulated by experience. One dimension of configurational knowledge which was assessed concerned distance knowledge. Interestingly, the symbolic distance effect, i.e., better performance on comparing distances with increasing distance differences, was stronger in participants who had lived for a long time in the city of Utrecht than for participants who had lived for a short time in the city of Utrecht. This effect of experience on correlation strength between interpair distance-ratios and RTs was, however, not different between males and females, nor were there any overall differences between males and females in correlation strength. These patterns indicate, in line with general notions on the development of spatial representations (see: Golledge, 1999) that the quality of environmental spatial representations in terms of metric features increases the longer individuals live in a new city. Males and females do not appear to differ in the quality of their spatial representation regarding distances within the city of Utrecht, nor in the speed with which this representational aspect has developed with experience.

Importantly, other elements of the spatial representations of Utrecht city did show gender effects. An experience by gender interaction was obtained for the distance comparison task when only considering trials in which distances for completely different place-pairs had to be compared, as opposed to trials in which place-pairs started with the same place. Apparently, experience may affect gender differences in configurational knowledge, depending on the way this knowledge is retrieved. RTs on the Distance comparison task showed that the former trials took more effort than the trials in which the starting place was repeated in the two distance pairs. This corroborates findings by Péruch et al. (2006). It has been suggested that males especially outperform females on difficult, cognitively demanding spatial tasks (Coluccia and Louse, 2004). The reason for an effect of cognitive load can be related to how males and females may differ in the way they retrieve information from memory. Males would be better in active mental manipulation or transformation of mental images, aspects which become more important when tasks get more complicated, whereas females excel in the use of static images (Vecchi and Girelli, 1998). Altogether, this suggests that with little experience, females may be less able to cope with high task demands.

Another measure of distance knowledge followed from the priming task. The advantage of this task is that performance is not affected by conscious cognitive processes, such as retrieval strategies, since participants are not aware of the actual aim of the task. This may be of particular value in examining gender effects where previous studies suggest that beliefs holding that spatial tasks are typically suited for males tasks and consequent thoughts on personal performance can negatively affect spatial achievements in females (Lawton, 1996; Moe and Pazzaglia, 2006). Moreover, priming tasks require a minimal cognitive load, which might lead to a purer assessment of spatial knowledge than more complicated, cognitively demanding tasks (Donald and Pellegrino, 1993). In previous studies, spatial priming has been shown to work for visually and verbally learned new environments (Denis and Zimmer, 1992; Noordzij and Postma, 2005). In the present study, spatial priming was also shown for well known places in a daily environment, which confirms and extends the symbolic distance effect. Primes close in space resulted in significantly faster RTs for the target than primes far in space. Both the symbolic distance effects and the priming effect indicate that places in the environment are spatially organized in the representation of the listener. Contrary to the symbolic distance effect, the spatial priming effect did not show an effect of experience. This might have to do with the fact that the symbolic distance effect taps into more continuous spatial distance measures, whereas the priming effect only concerns global or categorical proximity, i.e., near vs. far. Since metric characteristics take longer to develop, this might explain why a positive effect of experience was only shown for the symbolic distance effect. Nevertheless, experience affected general speed of recognition; participants who had lived for a short time in the city of Utrecht had slower RTs than participants who had lived for a long time in Utrecht. On itself it is an interesting finding that the present study showed a priming effect for well-known places in a daily environment. Apparently our thoughts automatically and unconsciously jump from one place to other places in the neighborhood.

Besides distance, another dimension of configurational knowledge was assessed: directional place knowledge. For the relative position task, in which the relative position of three places had to be verified, no effect of experience was found. However, now males outperformed females. Intriguingly, whereas part of the distance measures clearly were modulated by experience as well as gender, performance on the relative position task only seemed to be affected by gender. One could speculate that in daily navigation distance knowledge is acquired in a direct, obligatory way by route-based environmental explorations, whereas directional position knowledge requires active construction and abstraction by the observer. It might be that since direction knowledge is not essential for daily navigation, extra experience does not greatly enhance performance on this task. Most importantly, the relative position task data revealed that men are notably better here. This dimension of configurational knowledge thus clearly is sensitive to gender differences.

Given the observation of differences in configurational knowledge between men and women, one might question what the underlying cognitive mechanisms are. For this reason, we also included two general spatial abilities tests and correlated these measures with the indices of configurational knowledge. Only for males who had lived for a long time in the city of Utrecht significant correlations were shown between performance on the configurational tasks and the general spatial abilities, whereas no relations were shown for males having shorter experience with the city. This might have to do with the fact that retrieval strategies get more constant when knowledge on locations is firmly embedded. Performance on the difficult part of the Distance comparison task related to CB, whereas performance on the relative positions task showed a significant correlation with CB as well as MR performance. In women only the easy distance trials related to MR performance. This is in line with findings by Bosco et al. (2004), who showed that orientating in an environment is more strongly related to visuo-spatial working memory in men than in women. For females, who had lived for a short time in Utrecht, performance on the easy distance trials did correlate with MR performance. This observation is harder to interpret. Perhaps for distance pairs starting with the same place, one route-distance could be ‘projected’ on the other by mentally rotating one route-line in order to directly compare the two distances.

Whereas response patterns in the Distance comparison task suggest that the quality of the representations regarding spatial distances was comparable in males and female, they do not necessarily preclude the possibility that males and females can differ in the characteristics of their spatial representations. In the spatial information processing literature, starting with Siegel and White (1975), two strategies/representations can be dissociated on which individuals rely when they represent an environment: route knowledge and survey knowledge. In route knowledge directions/turns and landmarks are sequentially processed in an individual-centered fashion, whereas survey knowledge implies the formation of a viewpoint-independent map of the environment. Males are suggested to be more prone to develop such a representation, since they have a preference to process geometrical cues, which form an important basis for the formation of a viewpoint-independent “bird’s eye view.” It could be that for relatively ‘easy’ tasks, route representations are sufficient to correctly compute configurational measures, whereas for more complicated tasks, a survey representation, in which configurational relations are already present, results in superior performance. Nevertheless, despite the fact that a route representation can lead to correct distance estimations, one would expect a route representation to elicit longer reaction times than a survey representation (Chabanne et al., 2003). No gender differences in reaction times were observed, however, consistent with the notion that performance differences in males and females were not based on different types of spatial representations. Future studies should further address the structure of spatial representations in males and females. It could be that with shorter experience than considered in the present study, clearer differences between males and females in the characteristics of underlying representations may emerge.

Taken together, the current results reveal marked configurational knowledge differences between men and women. These differences are partly modulated by experience and are clearest for more difficult tests of distance knowledge. Interestingly, with time we obtain a more precise metric sense of where places are in our surroundings. Knowledge on relative positions on the other hand, is not enhanced by experience, but males do outperform females on this type of knowledge. Hence, the construction of a spatial representation in all its facets appears to depend on a mix of experience linked to a particular environment and either hardwired or developmental cognitive processing abilities.

## Conflict of Interest Statement

The authors declare that the research was conducted in the absence of any commercial or financial relationships that could be construed as a potential conflict of interest.
